# Stillbirth and neonatal death rates across time: the influence of pregnancy terminations and birth defects in a Western Australian population-based cohort study

**DOI:** 10.1186/s12884-016-0904-1

**Published:** 2016-05-17

**Authors:** Brad M. Farrant, Fiona J. Stanley, Pia Hardelid, Carrington C.J. Shepherd

**Affiliations:** Telethon Kids Institute, University of Western Australia, P.O. Box 855, West Perth, 6872 WA Australia; Population, Policy and Practice Programme, University College London Institute of Child Health, 30 Guilford Street, London, WC1N 1EH UK; Research Department of Primary Care and Population Health, University College London, Rowland Hill Street, London, NW3 2PF UK

**Keywords:** Stillbirth, Neonatal death, Birth defects, Pregnancy terminations, High-income countries

## Abstract

**Background:**

The stillbirth rate in most high income countries reduced in the early part of the 20^th^ century but has apparently been static over the past 2½ decades. However, there has not been any account taken of pregnancy terminations and birth defects on these trends. The current study sought to quantify these relationships using linked Western Australian administrative data for the years 1986–2010.

**Methods:**

We analysed a retrospective, population-based cohort of Western Australia births from 1986 to 2010, with de-identified linked data from core population health datasets.

**Results:**

The study revealed a significant decrease in the neonatal death rate from 1986 to 2010 (6.1 to 2.1 neonatal deaths per 1000 births; *p* < .01), while the overall stillbirth rate remained static. The stillbirth trend was driven by deaths in the extremely preterm period (20–27 weeks; which account for about half of all recorded stillbirths and neonatal deaths), masking significant decreases in the rate of stillbirth at very preterm (28–31 weeks), moderate to late preterm (32–36 weeks), and term (37+ weeks). For singletons, birth defects made up an increasing proportion of stillbirths and decreasing proportion of neonatal deaths over the study period—a shift that appears to have been largely driven by the increase in late pregnancy terminations (20 weeks or more gestation). After accounting for pregnancy terminations, we observed a significant downward trend in stillbirth and neonatal death rates at every gestational age.

**Conclusions:**

Changes in clinical practice related to pregnancy terminations have played a substantial role in shaping stillbirth and neonatal death rates in Western Australia over the 2½ decades to 2010. The study underscores the need to disaggregate perinatal mortality data in order to support a fuller consideration of the influence of pregnancy terminations and birth defects when assessing change over time in the rates of stillbirth and neonatal death.

## Background

Reductions in early life mortality rank as one of the greatest global public health achievements of the last century [1]. The available data highlight that these advancements extend to the perinatal period, although the scale and pattern of mortality rate trends have differed between the prenatal and neonatal periods. Marked reductions in the stillbirth rate occurred in high-income countries during the 20^th^ century [2–4]. Analysis of the data from 11 high-income countries showed a rapid decline beginning in the late 1930s to early 1940s (when the stillbirth rate for births of 28 weeks or more gestation was between 25 and 45 per thousand births) through to the year 2000 (when the stillbirth rate was between 3 and 5 per thousand births) [5]. A subsequent analysis for 12 high-income countries showed the stillbirth rate (for births of 28 weeks or more gestation) levelling off or reducing more modestly between 2000 and 2008 with Norway having the lowest rate of 2.2 per thousand births [6]. In contrast, the neonatal death (babies aged 0–27 days) rate has continued to decrease in recent decades in high-income countries [7], building on earlier improvements [2, 3]. The UN Inter-agency Group for Child Mortality Estimation found that the neonatal mortality rate in developed regions of the world fell by 55 %, from a rate of 8 deaths per thousand livebirths in 1990 to 3 deaths per thousand livebirths in 2013 [8].

Better quality data can disaggregate to enable a more nuanced picture of mortality trends and reasons for change. Since the middle of last century, stillbirths and neonatal deaths have typically been reported together as perinatal deaths [9,10]. More recently it has been argued that the divergence in the causes of stillbirths and neonatal deaths means that, particularly in high-income countries, they should be reported separately [9]. However, continued overlap in some of the causes indicates that there is still some benefit in assessing stillbirths and neonatal death rates together [10].

When assessing the change over time in the stillbirth and neonatal death rates for high-income countries it is important to consider the influence of terminations of pregnancy. This is because legislative changes and a higher coverage of prenatal ultrasound screening have led to increases in the number of terminations performed in many jurisdictions in recent decades [11–13]. In Western Australia, for example, terminations of pregnancy have been legally permissible since 1998. In addition, first trimester pregnancy screening has generally been available in Western Australia since 1999–2000, and has offered a more accurate set of tests for the detection of birth defects in the developing fetus [11–14]. Accordingly, research has found that pregnancy terminations associated with birth defects explained about two-fifths of the decrease in the total population mortality rate to 1 year of age in Western Australia between 1980 and 1998 [15]. The only study to investigate the contribution of pregnancy terminations on the stillbirth rate (in Northern England) found that increases in the proportion of stillbirths that were terminations of pregnancy raised the stillbirth rate by nearly 10 % between 1994 and 2005 [11]. It did not investigate whether the increasing pregnancy termination rate was associated with change in the neonatal mortality rate.

Another important issue to consider is the elevated stillbirth [16,17] and neonatal death [18] rates associated with increasing rates of multiple pregnancies in high-income countries since the 1970s, due to increased maternal age and assisted reproductive technologies [19, 20]. More recent changes to recommendations for assisted reproductive techniques have been associated with increasing rates of twin pregnancies and decreasing rates of higher order pregnancies [21]. Given the added risk factors associated with multiple births it is important to consider stillbirth and neonatal death rates separately for singleton and multiple births in order to avoid confounding what are potentially quite different trends. To our knowledge no previous research has simultaneously investigated the effects of pregnancy terminations and birth defects on the stillbirth and neonatal death rates for singleton and multiple births.

The purpose of the current study was to use linked Western Australian administrative data for the years 1986–2010 to investigate whether the overall stillbirth and neonatal death rates follow the trends observed in other high-income jurisdictions, to explore differences between singleton and multiple births, and to assess the influence of pregnancy terminations and birth defects on these trends.

## Methods

### Study population and data sources

This retrospective cohort study included all births with a gestational age of 20 weeks or more in Western Australia between 1986 and 2010, inclusive. Study data were primarily sourced from core population health datasets held by the Data Linkage Branch of the Western Australian Government Department of Health (DLB)—including the Midwives’ Notification System (MNS), Western Australian Register of Developmental Anomalies (WARDA), and the Birth Registration and Death Registration datasets (from the Western Australian Registry of Births, Deaths and Marriages). Information on birth outcomes (birth status: live birth or stillbirth, and gestational age) was sourced from the MNS and Births Registration datasets [15]. The MNS records the circumstances of all births of 20 weeks or more gestation, with information received from attending midwives. The WARDA includes all birth defects diagnosed at birth for stillbirths (including terminations of pregnancy) and livebirths as well as diagnoses for livebirths up to 6 years of age from a number of sources with a high level of ascertainment [22]. The Death Registration dataset was used to identify those in the cohort that had died.

These data were linked together by the DLB by probabilistic linkage using common identifiers including name, address and the birthdate of the baby [23]. Multiple linkage passes are conducted in order to minimise both false-positive and false-negative errors along with clerical review to resolve doubtful links. The procedures used in the extraction of data from the WA Data Linkage System (WADLS) have been internationally accepted as best practice [24] and the quality of linkages have been shown to be highly reliable [23]. The MNS record was used as the initial master file and data linked across datasets. Only de-identified data files were extracted (for each dataset) by the DLB and provided to the researchers [25]. We then merged the datasets using a linkage key.

### Defining stillbirth

In Australia, the accepted definition of stillbirth is a birth of a baby showing no signs of life, of at least 400 g birthweight or at least 20 weeks gestation. This is generally operationalised in epidemiological studies as 20 weeks or more and accords with the limits of the available data—this range (≥20 weeks) has been applied here (we did not have access to records of stillbirths of less than 20 weeks gestation of 400 g or more birthweight). Stillbirths were identified using the birth status variable on both the MNS and Death Registration datasets, with the small number of discrepancies (26 cases) resolved via consideration of other indicators of the circumstances of birth from these datasets and information on the cause of death. The birth status on the MNS was given priority in ambiguous cases. In Western Australia, terminations of pregnancy are included as stillbirths if they occur at 20 weeks or more gestation.

### Defining neonatal deaths

Neonatal deaths (death in the first 28 days of life) were identified using the information available in the Death Registration dataset in combination with the date of birth data from the MNS. The day of birth was nominated as day zero and in-scope neonatal deaths were identified as all non-stillbirth deaths with ages of 27 days or less.

### Terminations of pregnancy

Information on pregnancy terminations at 20 weeks or more gestation was obtained from the WARDA and the cause of death text field on the Death Registration record. In Western Australia, all termination procedures are notifiable under the Health Act 1911. While no upper gestational age limit is specified in the legislation, the vast majority of terminations are conducted prior to 20 weeks [26]. Late terminations (≥20 weeks) require approval by a panel of medical practitioners and are restricted to cases where there is a serious medical condition affecting the mother or fetus [27]. Terminations at this gestation are generally referred to as ‘late’ terminations and are managed and conducted in a structured manner in accordance with legislation [26]. The procedure involves the use of medication to terminate, i.e. is consistent with a medical (not surgical) abortion.

### Birth defects

The WARDA was used to identify children with birth defects, and codes cases according to the British Paediatric Association extension of the International Classification of Diseases Version 9. Only those with a diagnosis code that reflected a ‘major’ birth defect were classified as having a birth defect for the purposes of this study—this includes cases that require treatment and/or result in disability or loss of function. A full list of birth defects diagnosis codes is available on the WARDA website [28].

### Prematurity

Consistent with World Health Organization guidelines [29], extremely preterm birth was defined as occurring between 20 and 27 weeks gestation (inclusive), very preterm as 28 to 31 weeks, moderate to late preterm as 32 to 36 weeks, and term as 37 or more weeks.

### Statistical analysis

The stillbirth rate for each gestational age range was calculated as the number of stillbirths divided by the number of births in that particular gestational age range multiplied by one thousand. The neonatal death rate for each gestational age range was calculated as the number of neonatal deaths divided by the number of livebirths in that particular gestational age range multiplied by one thousand. Trends over time were analysed using linear regression, with the t-statistic used to assess statistical significance (regression coefficients (*β*s) are reported). Univariable logistic regression was used to assess the relative odds of a stillbirth and neonatal death between groups with and without birth defects (odds ratios reported). The two sampled *t*-test was used to assess whether average annual mortality rates were significantly different across population groups (*t* statistic reported). Results with *p* < .05 are regarded as statistically significant.

## Results

### Stillbirth and neonatal death rates: All births 1986–2010

For the 1986–2010 period there was a total of 657,540 births (337,003 male, 320,494 female, 43 undetermined gender) with a gestational age of 20 weeks or more, including 4707 stillbirths (7.2 stillbirths per 1000 births) and 2076 neonatal deaths (babies aged 0–27 days; 3.2 neonatal deaths per 1000 livebirths). Of these there were 652,381 births that had gestations of 28 weeks or more of which 2234 were stillbirths (3.4 stillbirths per 1000 births) and 1117 neonatal deaths (1.7 neonatal deaths per 1000 livebirths). Thus, over half of all recorded stillbirths and nearly half of all neonatal deaths occurred among the 0.8 % of births (5159) that occurred between 20 and 27 weeks gestation. The mortality rates were highest at this gestation, including stillbirths and neonatal deaths (479.4 per 1000 births and 357.0 per 1000 livebirths, respectively, across the study period).

**Fig. 1:**
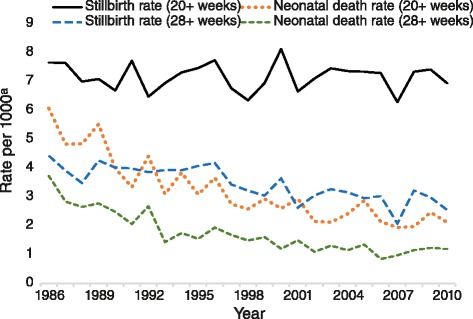
Stillbirth and neonatal death rate trends for all births of 20+ and 28+ weeks gestation. ^a^ Rate per 1000 births (stillbirths) or 1000 livebirths (neonatal deaths)

Figure 1 displays the stillbirth and neonatal death rates for all births of 20 weeks or more and 28 weeks or more gestation between 1986 and 2010. The stillbirth rate for all births of 20 weeks or more gestation did not significantly change over time (*β* = −0.08, *p* = .71). In contrast, the stillbirth rate for all births of 28 weeks or more gestation (4.4 in 1986, 2.5 in 2010, per 1000 births) and the neonatal death rates for all births of 20 weeks or more (6.1 in 1986, 2.1 in 2010, per 1000 births) and 28 weeks or more (3.7 in 1986, 1.2 in 2010, per 1000 births) gestation all significantly decreased over time (all *β’s* < −0.83, all *p’s* < .01).

### Singleton and multiple births

Multiple births accounted for almost 3 % (or 19,077 births; 9634 male, 9438 female, 5 undetermined) of all births of 20 weeks or more gestation, although comprised 9 % of stillbirths (or 443) and 19 % of neonatal deaths (or 389). There were 9157 pairs of twins, 235 sets of triplets and 14 sets of higher-order births. The average annual stillbirth rate for multiple births (23.6 per 1000 births) was significantly higher (*t* = 16.1, *p* < .01) than that for singleton births (6.7 per 1000 births). Similarly, the average annual neonatal death rate for multiple births (21.8 per 1000 livebirths) was significantly higher (*t* = 8.2, *p* < .01) than that for singleton births (2.7 per 1000 livebirths). Consistent with findings from other high-income jurisdictions, multiple pregnancy and twin pregnancy rates have significantly increased with time (all *β’s* > 0.59, all *p’s* < .01) with both appearing to peak in 2001 (1.8 and 1.7 %, respectively) while the higher-order pregnancy rate has significantly decreased over time (*β* = −0.43, *p* = .03) with a maximum of 0.1 % in the year 1989 and a minimum of 0.01 % in 2010.

As with the stillbirth rate for all births of 20 weeks or more gestation, the stillbirth rate for singleton births of 20 weeks or more gestation did not significantly decrease between 1986 and 2010 (*β* = 0.05, *p* = .82). In contrast, the stillbirth rate for multiple births of 20 weeks or more gestation (26.8 in 1986, 21.4 in 2010) and the neonatal death rates for singleton births (5.0 in 1986, 1.8 in 2010) and multiple births (49.5 in 1986, 12.1 in 2010) of 20 weeks or more gestation all significantly decreased over time (all *β’s* < −0.57, all *p’s* < .01).

### Trends by gestational age

The distribution of singleton births by gestational age has shifted significantly over the study period with the proportion of births at term (max 94.4 % in 1986, min 92.8 % in 2006) significantly decreasing (*β* = −0.94, *p* < .01) and the proportion of births at moderate to late preterm (min 4.3 % in 1988, max 5.8 % in 2010) and extremely preterm (min 0.5 % in 1990, max 0.8 % in 2009) significantly increasing across time (all *β’s* > 0.83, all *p’s* < .01) (the proportion of very preterm singleton births did not change significantly). For singleton births, the distribution of stillbirths and neonatal deaths by gestational age has also shifted quite markedly over this period. The proportion of stillbirths associated with extreme prematurity (20–27 weeks gestational age) increased significantly (42.9 % in 1986 to 62.8 % in 2010; *β* = 0.87, *p* < .01) across time whereas the proportion of stillbirths associated with very preterm (28–31 weeks) (11.3 to 8.0 %; *β* = −0.45, *p* = .03), moderate to late preterm (32–36 weeks) (16.7 to 9.0 %; *β* = −0.71, *p* < .01), and term births (37+ weeks) (29.2 to 20.1 %; *β* = −0.74, *p* < .01) have all significantly decreased across time. The proportion of neonatal deaths associated with extreme prematurity also increased significantly (*β* = 0.41, *p* < .05). However, the proportion of neonatal deaths associated with very preterm (*β* = −0.31, *p* = .13), moderate to late preterm (*β* = −0.29, *p* = .16), and term births (*β* = −0.13, *p* = .53) did not significantly change across time (Table 1).

**Table 1:** Time trends in the distribution of singleton births, stillbirths and neonatal deaths by gestational age

	Gestational age									
	20–27 weeks (extremely preterm)		28–31 weeks (very preterm)		32–36 weeks (moderate to late preterm)		37+ weeks (term)		Total	
	No.	%	No.	%	No.	%	No.	%	No.	%
Births										
1986	141	0.6	155	0.7	1001	4.3	22031	94.4	23328	100.0
1998	159	0.6	167	0.7	1285	5.2	23305	93.5	24916	100.0
2010	228	0.7	173	0.6	1780	5.9	28233	92.8	30414	100.0
*Mortality—*										
Stillbirths										
1986	72	42.9	19	11.3	28	16.7	49	29.2	168	100.0
1998	71	49.0	24	16.6	24	16.6	26	17.9	145	100.0
2010	125	62.8	16	8.0	18	9.0	40	20.1	199	100.0
Neonatal deaths										
1986	38	32.5	8	6.8	22	18.8	49	41.9	117	100.0
1998	25	39.7	4	6.3	5	7.9	29	46.0	63	100.0
2010	21	37.5	4	7.1	8	14.3	23	41.1	56	100.0

**Fig. 2:**
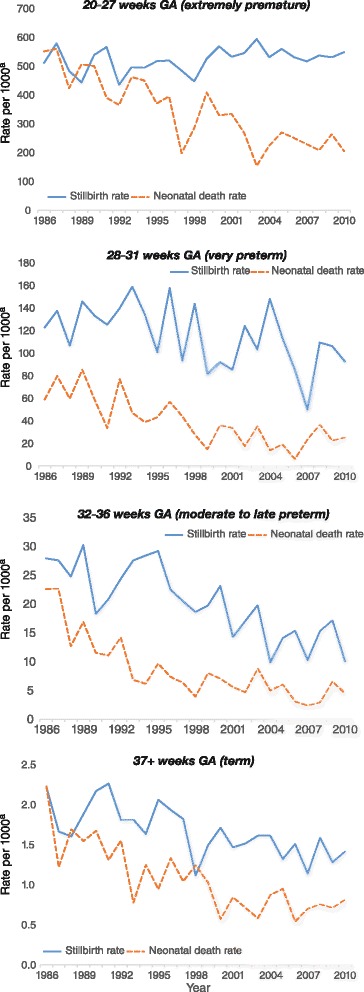
Stillbirth and neonatal death rate at each gestational age range for singleton births. ^a^ Rate per 1000 births (stillbirths) or 1000 livebirths (neonatal deaths). *GA* Gestational age

Furthermore, the stillbirth rate associated with singleton babies born extremely premature has shown a non-significant increase (*β* = 0.34, *p* = .10) whereas the stillbirth rate for very preterm (*β* = −0.51, *p* < .01), moderate to late preterm (*β* = −0.81, *p* < .01), and term births (*β* = −0.69, *p* < .01) have all significantly decreased over time (see Fig. 2). In contrast, the neonatal death rate decreased significantly (all *β’s* < −0.78, all *p’s* < .01) over time across all these gestational age groups (see Fig. 2).

### Pregnancy termination rates

Our analysis identified 796 pregnancy terminations at 20 weeks or more gestation between 1986 and 2010 (402 male, 385 female, 9 undetermined gender). There were no pregnancy terminations involving triplet, quadruplet or quintuplet pregnancies with 29 involving twin pregnancies (1.6 pregnancy terminations per 1000 births) and 767 involving singleton pregnancies (1.2 pregnancy terminations per 1000 births).

There has been an almost 4-fold increase in the pregnancy termination rate for singleton pregnancies of 20 weeks or more gestation between 1986 and 2010 (from 0.5 to 1.9 per 1000 births; *β* = 0.88, *p* < .01). The proportion of stillbirths that were terminations of pregnancy also significantly increased (*β* = 0.89, *p* < .01) across time.

**Fig. 3:**
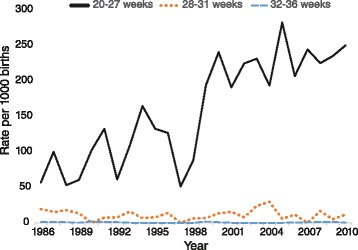
Pregnancy termination rate by gestational age for singleton pregnancies

As expected, the vast majority of pregnancy terminations of post-19 week singleton pregnancies occurred at 20–27 weeks gestation (709 of 767). Smaller numbers were recorded at 28–31 weeks (42), and 32–36 weeks (16), with none at 37 weeks or more gestation. As can be seen in Fig. 3, there was a significant increase in the termination rate associated with extreme prematurity (*β* = 0.86, *p* < .01), with about a quarter of births at this gestational age being terminations. The slight decrease in the rates at very preterm and at moderate to late preterm were not significant (both *β’s* > −0.19, both *p’s* > .37).

**Fig. 4:**
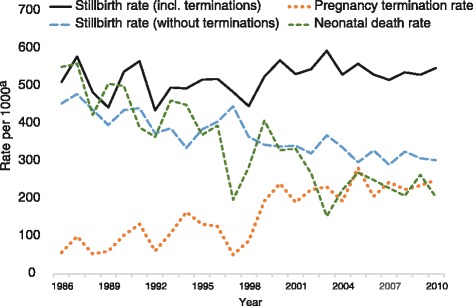
Stillbirth rate with and without pregnancy terminations for singleton births 20–27 weeks gestation. ^a^ Rate per 1000 births (stillbirths, terminations) or 1000 livebirths (neonatal deaths)

Indeed, when pregnancy terminations are excluded, the stillbirth rate for extremely preterm singleton births significantly decreases (*β* = −0.87, *p* < .01) over time (see Fig. 4). Thus, it appears that the discrepant trends for the stillbirth rate at differing gestational ages (extremely preterm non-significantly increasing vs other gestational age periods significantly decreasing) is at least partially a result of increased pregnancy terminations in the extremely preterm period. For extremely preterm singleton births, the increase in the pregnancy termination rate across time is significantly negatively correlated (*r* = −.63, *p* < .01) with the decrease in the neonatal death rate with the variation in the former explaining 36 % of the variation in the latter.

### Birth defects

Across the 1986–2010 period, there were 36,490 births (21,217 male, 15,253 female, 20 undetermined gender) of 20 weeks or more gestation reported to have had a birth defect(s). Of these 30,122 (82.5 %) were reported as having a major birth defect(s) (17,999 male, 12,103 female, 20 undetermined gender) which are the focus of the following analyses. There were no reports of a major birth defect(s) involving quadruplet or quintuplet pregnancies with 52 involving triplet pregnancies (73.8 cases per 1000 births), 1079 involving twin pregnancies (58.9 cases per 1000 births) and 28,991 involving singleton pregnancies (45.4 cases per 1000 births). The proportion of singleton, twin and triplet births with major birth defects has not changed significantly (all *β’s* > −0.27, all *p’s* > .20) across time.

**Fig. 5:**
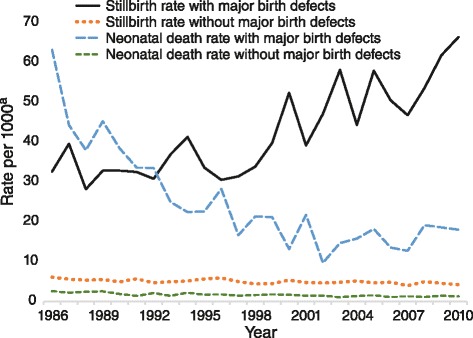
Stillbirth and neonatal death rates for singleton births with and without major birth defects. ^a^ Rate per 1000 births (stillbirths) or 1000 livebirths (neonatal deaths)

Across the 1986–2010 period, the stillbirth rate for singleton babies with major birth defect(s) was 42.3 per 1000 births and for twins with a major birth defect(s) it was 39.8 per 1000 births (none of the 52 triplets with major birth defect(s) was stillborn). The neonatal death rate for singleton babies with a major birth defect(s) was 24.7 per 1000 livebirths, for twins with a major birth defect(s) it was 57.9 per 1000 livebirths, and for triplets with a major birth defect(s) it was 19.2 per 1000 livebirths. Compared to singleton babies without a major birth defect(s), singleton babies with a major birth defect(s) had a 781 % increased risk of stillbirth (*p* < .01) and a 1377 % increased risk of neonatal death (*p* < .01). However, the stillbirth rate for singleton babies with a major birth defect(s) has increased (*β* = 0.83, *p* < .01) across time whereas the stillbirth rate for singleton babies without a major birth defect(s) and the neonatal death rate for singleton babies with as well as those without a major birth defect(s) have all significantly decreased (all *β’s* < −0.67, all *p’s* < .01) across time (see Fig. 5).

However, for singleton pregnancies of 20 weeks or more gestation, the proportion of stillbirths involving major birth defects has significantly increased (*β* = 0.89, *p* < .01) over time while the proportion of neonatal deaths involving major birth defects has significantly decreased (*β* = −0.67, *p* < .01) over time. Indeed the two are significantly negatively correlated (*r* = −.72, *p* < .01) with the variation in the proportion of stillbirths involving major birth defects explaining 50 % of the variation in the proportion of neonatal deaths involving major birth defects.

These findings suggest that a significant proportion of perinatal deaths involving major birth defects have shifted from the neonatal period to the pregnancy/birth period (stillbirths) between 1986 and 2010. Indeed, the proportion of singleton pregnancies (of 20 weeks or more gestation) with major birth defects that were pregnancy terminations has significantly increased (*β* = 0.89, *p* < .01) over time and this increase is significantly positively correlated (*r* = .90, *p* < .01) with the increase in the proportion of stillbirths involving major birth defects (*R*^2^ = 0.80). Thus the shift in perinatal deaths involving major birth defects from the neonatal period to the pregnancy/birth period (stillbirths) appears to have been largely driven by the increase in terminations of pregnancy. Indeed, when pregnancy terminations are excluded, the proportion of stillbirths involving major birth defects shows a significant decrease over time (*β* = −0.42, *p* = .04). Of the terminations of singleton pregnancies (of 20 weeks or more gestation) with major birth defect(s), 92.5 % (701) occurred in the extremely preterm period, 5.5 % (42) occurred in the very preterm period, and 2.0 % (15) occurred in the moderate to late preterm period.

Across the 1986–2010 period, when compared with twins without major birth defect(s), twins with major birth defect(s) had a 97 % increased risk of stillbirth (*p* < .01) and a 249 % increased risk of neonatal death (*p* < .01). The stillbirth rate for twins with major birth defect(s) has not significantly changed (*β* = −0.12, *p* = .56) across time whereas the stillbirth rate for twins without major birth defect(s) and the neonatal death rate for twins with as well as those without major birth defect(s) have all significantly decreased (all *β’s* < −0.48, all *p*’s < .02) across time. However, for twins, the proportion of stillbirths involving major birth defects has not significantly changed over time (*β* = 0.02, *p* = .92) nor has the proportion of neonatal deaths involving major birth defects (*β* = 0.06, *p* = .77).

## Discussion

The study highlights that there has been a steady reduction in the stillbirth rate in Western Australia (from 1986 to 2010) for pregnancies that ended in birth at or after 28 weeks gestation, but no discernible change in the rate at 20–27 weeks gestation. In contrast, there has been a consistent decline in neonatal mortality over this period. We suggest that part of the decrease in risk of stillbirth at later gestations and neonatal death is due to a shift in mortality to earlier gestational periods.

These trends are broadly consistent with those reported in other high-income jurisdictions [5–7], although there are discernible differences. The overall stillbirth rate trend in the United States for births of 20 weeks or more gestation, for example, is characterised by marked reductions in the 1970s and 1980s with more modest improvements in the 1990s and early 2000s [4,30]. In contrast, the overall stillbirth rate in Western Australia did not significantly change across the 1986–2010 period, with the average rate for births at or after 20 weeks gestation across the study period (7.2 per 1000 births) being higher than that observed in the United States in 2003 (6.2). Although differences in definitions and inclusions as well as data quality and availability need to be kept in mind, these findings suggests that further reductions in stillbirth are possible.

There has been a significant increase in the rate of late pregnancy terminations (post-19 weeks gestation) in Western Australia—particularly from the mid-1990s—and this was primarily driven by terminations at 20–27 weeks gestation. Very few studies have investigated the contribution of pregnancy terminations to the stillbirth rate. Our evidence is consistent with the findings of a previous study in Northern England, and indicates that terminations of pregnancy account for an increasing proportion of stillbirths across time [11]. This reflects the legalisation of late termination of pregnancy in Western Australia from 1998 specifically, and the higher coverage of prenatal screening and improvements in prenatal diagnosis techniques in recent decades [11–13]. Our findings also show that pregnancy terminations: (1) at least partially account for the discrepant trends in the stillbirth rate at different gestational ages (a small, marginally significant increase at 20–27 weeks vs significant reductions at all other gestational age periods); and (2) explain over a third of the variation (decrease) in the neonatal death rate over time for extremely preterm singletons.

Identifying babies with and without a major birth defect(s) has provided a greater understanding of the reasons for the shift in the composition of perinatal mortality over time in Western Australia. The study has linked routine information on birth and death circumstances to a robust register of developmental anomalies, and highlighted that not only do singleton babies with a major birth defect(s) have a profoundly greater chance of being stillborn (than those without a birth defect) but that the risk has increased over time. This trend is in stark contrast to the stillbirth rate for singleton babies without a major birth defect(s) and the neonatal death rate for singleton babies with and without major birth defect(s)—which have all significantly decreased. Indeed, the variation (increase) in the proportion of stillbirths involving major birth defects explained 50 % of the variation (decrease) in the proportion of neonatal deaths involving major birth defects.

While not empirically conclusive from our data, the study findings suggest that changes in clinical practice related to pregnancy terminations has played a substantial role in shaping stillbirth and neonatal death rates over time. Some of the decrease in risk of neonatal death and stillbirth risk for births at or after 28 weeks gestation across the study period is likely the result of a shift in mortality to earlier gestational periods. This shift appears to have been largely driven by an increase in the termination of pregnancies (at 20–27 weeks) with a diagnosed major fetal abnormality. It is important to note that the scope of the current study is, to a degree, limited by available data. The MNS excludes information on births that occurred before 20 weeks gestation, which is the period when the vast majority of terminations are performed [15,31]. It is likely that the trends in stillbirth and neonatal death rates observed in the current study were influenced by pregnancy terminations occurring before 20 weeks gestation.

The study confirms that the proportion of multiple pregnancies increased through the 1980s and 1990s (appearing to peak in 2001), as has the likelihood of these babies surviving the perinatal period—these findings are broadly consistent with trends in other high-income jurisdictions [19–21]. The trends in stillbirth and neonatal deaths over time, however, are more complicated for multiple than singleton births. While the overall stillbirth and neonatal death rates for multiple births significantly decreased over time, there has been no change in the stillbirth rate for twins with a major birth defect(s).

The present study underlines the importance of disaggregating mortality data by stage and age of death and plurality. This enables a much more nuanced picture of the trends in mortality and helps identify trends that can be masked by a headline perinatal death rate figure. Despite the difficulties in accurately measuring gestational age [32], the current results have highlighted that there are substantial differences in the mortality trends at different gestational ages. These differences are more clearly understood by considering, inter alia, the effects of changes in obstetric practice related to terminations of pregnancy. Although they were not the focus of the current study, clearly the consideration of a broader range of maternal and other risk factors will support a more accurate understanding of mortality patterns, underlying causes, and opportunities for prevention. This ambition needs to be matched by the capacity of the available data. Linked administrative data, such as that used in this study, provides a population-representative source that can support an analysis of trends with the consideration of changes in clinical practice, socio-demographics and other important risk factors. This, in turn, can assist in a more active discourse on the reasons for changes in preventable perinatal mortality.

Up to two-thirds of stillbirths in high-income countries continue to be classified as unexplained [33–35]. In Western Australia, 25 % of stillbirths in 2004–2008 were classified as of unknown cause and 37 % as caused by extremely low birthweight (<1000 g) [36]. International research has found that over half of ‘unexplained’ stillbirths are associated with Intrauterine Growth Restriction (IUGR) [34,37]. A recent meta-analysis for high-income countries found that IUGR quadrupled the risk of stillbirth (adjusted OR = 3.9, 95 % CI: 3.0–5.1) with a population-attributed risk of 23.3 % [38]. Indeed, McKenna and Dornan (2005) [39] argued that the term ‘unexplained stillbirth’ should be replaced with ‘unpredicted stillbirth often due to IUGR.’ In the next phase of our research we will use linked administrative data to further investigate the trends in antepartum and intrapartum stillbirth and neonatal death risk profiles utilising data on a broad range of risk factors. This will include IUGR, assessed both with population-derived gestation specific cut-offs and customised cut-offs based on maternal/parental characteristics (particularly when IUGR occurs in the absence of birth defects), pregnancy and labour complications, and maternal age and ethnicity.

## Conclusions

The trends observed in Western Australian stillbirth and neonatal death rates over the years 1986–2010 in the current study are broadly consistent with those observed in other high-income jurisdictions. The stillbirth and neonatal death rates for all births of 28 weeks or more gestation and the neonatal death rate for all births of 20 weeks or more gestation all significantly decreased while the stillbirth rate for babies of 20 weeks or more gestation did not change significantly. Over half of all recorded stillbirths and nearly half of all neonatal deaths occurred among the 0.8 % of births that occurred between 20 and 27 weeks gestation. For singletons, the proportion of stillbirths involving major birth defects has significantly increased over time while the proportion of neonatal deaths involving major birth defects has significantly decreased with the results indicating that this shift appears to have been largely driven by the increase in pregnancy terminations at 20 weeks or more gestation. Our results highlight the importance of disaggregating perinatal mortality data to support a fuller consideration of the influence of pregnancy terminations and birth defects when assessing change over time in stillbirth and neonatal death rates.

## Ethics approval and consent to participate

This research was granted ethics approval by the Western Australian Department of Health Human Research Ethics Committee (#2011/64) and the Western Australian Aboriginal Health Ethics Committee (#613). These ethical approvals support a waiver of consent on the basis that the study: (1) utilises routinely collected information from existing administrative datasets (and, accordingly, does not include active participants); and (2) only has access to de-identified data, which are stored, analysed and disseminated according to strict protocols.

## Consent for publication

Not applicable.

## Availability of data and materials

The authors do not have permission to share the data used in this project, which were provided by the Data Linkage Branch of the Western Australian Government Department of Health under strict conditions.

## Abbreviations

DLB: Data Linkage Branch of the Western Australian Government Department of Health
IUGR: Intrauterine Growth Restriction
MNS: Midwives’ Notification System
WADLS: Western Australian Data Linkage System
WARDA: Western Australian Register of Developmental Anomalies
